# Access to historic individually identifiable health information: a multi-institutional survey of research institutions

**DOI:** 10.5195/jmla.2026.2259

**Published:** 2026-07-01

**Authors:** Amanda Garfunkel

**Affiliations:** 1 amg4018@med.cornell.edu, Digital Archivist, Medical Center Archives, Samuel J. Wood Library and C.V. Starr Biomedical Information Center, Weill Cornell Medicine, New York, NY

**Keywords:** Access to Information, Historical Research, Privacy, Medical Collections, Medical Humanities

## Abstract

**Objective:**

Access to historic individually identifiable health information has been studied from a researcher perspective, but there has not been a comprehensive review of access policies at an institutional level to see if there is any consistency across institutions. The objective of this study is to gather preliminary information on these access policies and potentially identify similarities and gaps to inform future research.

**Methods:**

This study employs an online survey for data collection about respondents' access policies and procedures at their institution. The final survey instrument consisted of 29 questions with a mix of multiple choice and free-text questions. The survey was distributed through listservs and posted in forums that targeted qualified participants.

**Results:**

The survey received 36 responses, 21 of which met the criteria for inclusion in analysis. Some notable results are that covered entities are nearly equally split on when to open research collections, non-covered entities have a wide range of access policies, and all respondents were nearly equally split on how long it has been since they last updated their access policy.

**Conclusion:**

While the number of responses was low for this study, the results identified areas that would benefit from further research and more robust study methods to potentially achieve a more standardized approach to access policies at research institutions.

## INTRODUCTION

Access to individually identifiable health information (IIHI) is legally regulated for modern medical records and medical information to protect the privacy of individuals. The Health Insurance Portability and Accountability Act (HIPAA) was enacted in 1996 and established a framework for access to this information, which is foundational knowledge for those who work with IIHI [1]. There are also research collections holding materials containing historic IIHI, outside the legal limit of HIPAA, that can be found in medical libraries with special collections, archives, and museums. These research collections contain primary source materials that document the history of medicine, offering opportunities for longitudinal views of medical practice. Some examples of these materials may include a 100-year-old medical register, antique medical instruments, research from prominent physicians, or more recent medical records among other things. However, for those who wish to access and use historic IIHI for their research, they often encounter a patchwork of legal and institutional barriers that can lead to confusion, frustration, and ultimately lack of access.

Research institutions that provide access to historic IIHI grapple with various competing priorities, which include: the changing legal landscape, ethical concerns, status as a covered entity (defined as an individual or organization that conducts electronic healthcare transactions and is subject to HIPAA rules) [2], and additional requirements from parent institutions. Between the enactment of HIPAA in 1996 and the subsequent Privacy Rule of 2003 [3], many institutions restricted access to their research collections containing IIHI as they determined whether they were considered a covered entity and to comply with the privacy rule that stipulated IIHI be protected in perpetuity [4][5]. Archivists testified to the National Committee on Vital Statistics in 2005 in support of restriction clarifications that were eventually included in the Health Information Technology for Economic and Clinical Health Act, also known as the HITECH Act [6], which amended the privacy rule to say that IIHI is no longer covered by HIPAA fifty years after a person’s date of death [7]. Beyond the legality of access to historic IIHI at the federal level, research institutions may also have to contend with additional restrictions at the state level. Understandably, leadership at these research institutions, particularly those at covered entities, have often implemented additional restrictions or longer timeframes to access IIHI beyond the legal limitations.

The changes in privacy laws and disparities between covered and non-covered entities have created confusion and challenges as librarians and archivists attempt to facilitate access to students and researchers who wish to utilize research collections containing historic IIHI. A grant funded by the Council of Library and Information Resources (CLIR) allowed two institutions, the Francis A. Countway Library of Medicine and The Alan Mason Chesney Medical Archives of the Johns Hopkins Institutions, to make available public health collections as well as conduct discussion sessions and launch a survey to gain a better understanding of challenges researchers encountered when trying to access collections that contain IIHI. The results of the project showed that many researchers never attempted to gain access to the research collections because the approval process was either too long, or too complicated, or they were skeptical they would be granted access in the end after investing so much time and energy into the application process. The project resulted in published recommendations for libraries and archives to enhance the description of research collections, so that students and researchers know what kind of IIHI the records contain to better determine if it is worth their effort to go through the process of gaining access, as well as develop clear and transparent access policies [8].

In the past decade, there have been multiple case studies and project-specific articles about the challenges of providing access to collections containing historic IIHI [9,10], but there hasn’t been a comprehensive review of access policies at an institutional level. The view from the researcher’s perspective is well documented from the previously mentioned CLIR project, but to date, there has not been a survey from the institutional side for medical libraries, archives, and museums to see if there is any consistency across institutions. Research objectives for this study included determining whether there are any similarities in the way research institutions provide access to IIHI, if there are any differences between access policies at covered and non-covered entities, and if any gaps emerge that can guide future research.

## METHODS

### Study Design

The study aimed to gain a preliminary understanding of current access policies related to historic IIHI and to potentially identify trends on consistency across institutions. The study was conducted virtually by creating an online survey that was designed to be completed by individuals who routinely provide access to their institution’s research collections. Survey questions utilized a combination of multiple choice and free text answers to capture professional demographic information, geographic location information, current institutional procedures for accessing historic IIHI, and current practices to implement those procedures. The survey instrument was created with a focus on gathering information related to the primary research objectives. Questions were written to collect data on the type of institution the respondents were affiliated with and details about their access policies. The instrument was modeled after similar surveys addressing privacy and staffing such as those written by Windon and Tang [11], Hawk [12], the National Digital Stewardship Alliance [13], and the Society of American Archivists [14]. The instrument went through revisions based on feedback received from colleagues to enhance question clarity and directness. Modifications were made to survey questions to attempt to gather information on the many variables within access and use policies for research collections. This study was approved by the Weill Cornell Medicine Institutional Review Board (IRB Record: 24-03027248) in May 2024.

The survey instrument itself was developed in an institutional Qualtrics account and consisted of 29 questions [15], however, skip logic was used so that participants could answer questions that most closely aligned with their situation and did not have to review every question. The largest differentiation being that survey respondents were shown two separate sets of questions depending on if they were affiliated with a covered entity or not. Survey respondents who indicated they were affiliated with a covered entity were asked questions about their access policies for collections during and after HIPAA regulations. Those who indicated they were not affiliated with a covered entity were asked about their overall access policies. All respondents were asked about their demographic and geographic information and their reference practices. The survey was made available for two months to collect responses from participants. During that time, any data that was collected was stored within Qualtrics until it could be exported for analysis.

### Participants

The survey was distributed through several listservs and discussion forums (including Medical Library Association; Librarians, Archivists, and Museum Professionals in the History of Health Sciences; Society of American Archivists’ Science, Technology, and Health Care section; Society of American Archivists’ Privacy and Confidentiality Section) to recruit participants who are affiliated with medical libraries, archives, or museums. Participation was limited to those who are based in United States institutions as international laws over medical records are out of scope for this study. The only other requirements for participation were that the respondent needed to be affiliated with an institution that holds research collections that contain historic IIHI and is somehow involved in navigating access to these collections for students and researchers. All participants were shown a consent statement before beginning the survey and all questions were left optional to encourage participation and protect privacy. Participants were free to stop the survey at any time. The goal for this preliminary survey was to get 25 respondents. This goal was established based on the number of respondents to a survey on the researcher’s perspective which received 63 responses. Assuming that there are multiple researchers responding on their experience for a single repository, the expectation is that there would be less responses for a survey focusing on the institutional perspective.

### Data Analysis

Analysis of the survey responses used a combination of quantitative and qualitative methods to gather results. The convenience sample produced by the survey responses utilized descriptive statistics to summarize responses in the demographic and geographical sections of the survey [16]. The data was able to be exported to a spreadsheet to perform the quantitative analysis and generate frequency distributions from individual questions. Free text questions utilized a content analysis method where certain words, phrases, themes, or concepts are identified and given a label [17]. Free-text responses that received the same content analysis label were then able to be analyzed and a frequency distribution produced. Combining these two methods allowed for a granular breakdown of results for each individual question to find commonalities and consensus.

The high number of variables possible with each survey response and the larger number of free-text questions meant there needed to be some manual analysis of the data to identify themes. Manual manipulation of survey data included grouping responses from similar types of institutions, similar use practices, and/or similar access restrictions together to attempt to identify common trends. For example, responses measuring the duration of access restrictions were grouped as those only restricting for the duration of legal timeframe, those who restrict for some amount of time after the legal timeframe, and those who restrict access permanently. The compiled themes were compared against other groupings to identify differences and areas of access policy that would benefit from further research.

## RESULTS

The results were calculated by using the completion rate of the responses instead of a response rate. It was not possible to calculate a response rate as there is no way of knowing how many individuals were on individual listservs or able to view the survey announcement in particular forums. A total of 36 respondents started the survey and 17 completed it for a completion rate of just over 47%. An additional 4 respondents completed 80% or more of the survey and were included in the analysis since the item nonresponses related to uses of identified IIHA but questions regarding access policies were completed [18]. This increased the number of responses from 17 to 21 meaning 58% of responses were included. The remaining 15 responses were excluded from analysis.

Of the included survey responses, about half of respondents (*N*=14 or 67%) were affiliated with academic research institutions. Other responses included medical (*N*=4 or 19%), public/governmental (*N*=2 or10%), museum (*N*=1 or 5%), and other (*N*=1 or 5%) types of research institutions. A majority of the survey respondents identified as an archivist (*N*=16 or 76%) while others identified as a curator (*N*=3 or 14%) or other (*N*=2 or 10%).

Characteristic details about the institutions the survey respondents are affiliated with are detailed in Table 1. The survey respondents were nearly unanimous (95%) in noting that their institutions have research collections that are open to the public. This means the collections are available to others outside of the immediate members of their primary institution and who may or may not have an institutional affiliation at all. Over half (*N*=12 or 57%) of survey respondents are affiliated with an institution that is a HIPAA covered entity. This means that these institutions are required to follow the federally regulated framework to access IIHI until 50 years after the person’s date of death. Interestingly, a large portion of survey respondents (*N*=9 or 43%) were unsure whether there was additional state laws related to the protection of IIHI indicating this may be an area requiring further investigation.

**Table 1:** Institutional Characteristic Survey Results

Characteristic	No. of Responses	%
Collections available to the public		
Yes	20	95%
No	1	5%
HIPAA Covered Entity		
Yes	12	57%
No	6	29%
Not Sure	2	10%
No Answer	1	5%
Additional State Laws		
Yes	6	29%
No	5	24%
Not Sure	9	43%
No Answer	1	5%

### Covered and non-Covered Entities

The distinction between what type of entity respondents were affiliated with decided what type of questions they were presented in the survey. The type of entity is important as covered entities are legally subject to HIPAA regulations where non-covered entities are not. Survey respondents who indicated they were affiliated with a covered entity were asked questions about what their access policies consisted of for HIPAA covered research collections, if there was a point in time when collections would be freely available for research (after the end of HIPAA), and what exceptions (if any) are made at any point during a research collection’s lifespan. Table 2 details the procedures and duration of access policies for covered entities.

**Table 2:** Covered Entities Access Summary

Access Summary	No. of Responses	%
Procedure to Access		
IRB Waiver	4	33%
Redaction	5	42%
Privacy Board	2	16%
Records Closed	2	16%
Duration of Restrictions		
End of HIPAA	5	42%
Set time after HIPAA	1	8%
Institutional Policy	1	8%
Always Require Approval	5	42%

Of the 12 survey respondents who were affiliated with covered entities, 5 (42%) said they redact collection records before providing access while they are still covered by HIPAA (redaction includes somehow obscuring the 18 identifiers outlined by HIPAA as well as other personal information), 4 (33%) require the researcher to obtain an IRB waiver, 2 (17%) require the researcher to get approval from an institutional privacy board, and 2 (17%) keep the collection records closed (unavailable for access) while they still fall under HIPAA. Two of the respondents utilized multiple procedures for access. When asked about the duration of restrictions after records are no longer covered by HIPAA, 5 (42%) of respondents said their institutions open up collections freely for research, 5 (42%) said the collection records always require prior approval (no matter the age of the collection record) before granting access, 1 (8%) said collection records are made available a certain number of years after the end of HIPAA restrictions, and 1 (8%) has a specific institutional policy beyond HIPAA.

There were 6 survey respondents who indicated they were affiliated with non-covered entities. Questions posed to these participants focused on gathering information about access requirements for their research collections. Answers to these questions showed there was no consensus on how to handle historic IIHI, however, respondents sometimes listed procedures used by participants at covered entities. One respondent noted that access to historic IIHI for deceased individuals is “somewhat of a judgement call.” Other procedures mentioned by the respondents include granting access only after a researcher obtains an IRB waiver and utilizing an institutional privacy board to approve researcher requests for access. One respondent noted that their policy for access states that materials over 100 years old are freely available, but anything less than 100 years requires the researcher to deidentify information.

### Other Services

This study also aimed to find out in what ways access to historic IIHI is delivered to researchers in addition to the institutional procedures. Redaction was mentioned in some respondents’ responses about access procedures and when asked directly 15 (71%) respondents said there are instances where they use redaction of collection materials to provide access to historic IIHI. Five respondents specifically mentioned creating redacted photocopies or using online software to redact digital records before allowing researcher access to collection materials. When asked if collections must be used by researchers onsite at the holding institution, 11 (52%) of survey respondents said ‘yes’ and three of those respondents said they must be viewed onsite but only after the end of HIPAA restrictions. When asked about providing remote reference services to researchers (remote reference services include email, phone, mail, etc.), 10 (48%) said they do provide remote reference services for collections containing historic IIHI.

**Figure 1:**
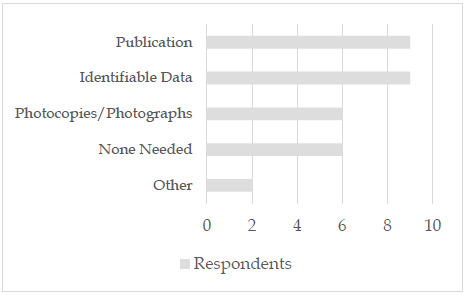
Uses of Collection Materials that Require Prior Approvals

There were two questions on the use and distribution of collection materials containing IIHI. Figure 1 details the results of the multiple-choice question asking respondents what uses of collection materials containing IIHI (of any age) require prior approval. Respondents were allowed to select as many uses as were applicable. Results showed that 9 (43%) require prior approval for publications, 9 (43%) require prior approval for identification of patient information, 6 (29%) need approval for photographs, 6 (29%) said no prior approvals are required after the end of HIPAA, and 2 (10%) marked other but failed to describe the circumstance. When asked about the digital availability of photographic material that contains patient’s faces, 11 (52%) said they do not make those images available online, 3 (14%) do make them available online after the end of HIPAA, and 5 (24%) had a different institutional policy. Finally, when asked how long ago their institutional access policy was updated 2 (10%) of respondents are in the process of updating their access policy, 5 (24%) updated their policy in the past 1-2 years, 4 (19%) updated their policy in the past 2-5 years, 1 (5%) updated their policy in the past 5-7 years, and 5 (24%) updated their policy over 7 years ago.

## DISCUSSION

This study provides some preliminary information on the access policies at research institutions whose collections contain historic IIHI and other types of medical records. The results offer a glimpse into various themes that can point to areas for further research and potential possibilities for standardizing access policies to decrease confusion among students and researchers and lower barriers to accessing research collections. Current users of these collections include descendants who have a legally outlined procedure to access materials, and researchers who have the capacity to go through complicated and varying procedures to gain access to historical records among others [19]. Medical researchers who wish to capture aggregate data from historic records for their research face lengthy timelines to access multiple sets of analog historical records [20]. The increase in digital medical records and changing laws to adapt to the technology means that there is potential for even more legal challenges, leading to increased restrictions and exacerbating an already confusing landscape [21].

The survey results suggest that institutions holding research collections may have a vested interest in making access policies and procedures easier to navigate for the staff handling research requests. Sixteen out of the twenty-one survey respondents have ten people or fewer working in their department, but nearly all have collections that are open to the public. Open research collections expand the availability of information but increase the amount of reference needed, which requires more staff time [22]. Institutions with a small staff may find this particularly taxing to manage reference requests and collection restrictions [23]. Furthermore, the lack of awareness on relevant state laws around privacy and health records from respondents could indicate that staff are not being equipped with the knowledge to handle these requests effectively and may close collections preemptively over an abundance of caution. Both of these areas are fertile ground for further research as staffing challenges and a changing information landscape are common themes within the library, archives, and museum professions.

Variances in survey responses also occurred between covered and non-covered entities. Research collections held at institutions that are considered covered entities are legally obligated to adhere to the HIPAA framework for access. Research collections held at non-covered entities do not have the same legal requirements, and it is often left to the discretion of the institution to decide how and when to open research collections for access.

This study indicated that there may be a wide spectrum of practices among non-covered entities despite some incorporating some of the same practices mentioned by respondents at covered entities. It was undeterminable what accounted for these wide variances. Without a legal obligation were restrictions (if any) implemented on strictly ethical grounds? Is it easier to take the lead from institutions who have a legal mandate? The rationale behind current access policies at non-covered entities is an area that would benefit from further research. The respondents from covered entities were nearly split on how restrictions are handled after the end of legal regulations. Some respondents open collections to be freely available for research after the end of HIPAA, while others keep collections restricted permanently and always require prior approval for access to collection material. Permanently restricted materials can particularly cause confusion as some students and researchers don’t understand why information, that is sometimes hundreds of years old, requires advanced approval for access. This is an area where standardization would be beneficial among all entities. Creating and advocating for a standard practice that can then be submitted to an institution’s counsel or privacy board would greatly reduce a barrier of access and lighten the workload for staff managing the access requests.

This study also aimed to capture data on the practices employed when providing reference services to students and researchers who request to view collection materials that contain historic IIHI. Questions were asked about how reference services are carried out, where collection materials are available to be viewed, if redaction is a tool that is ever employed to make materials accessible, and if materials are ever made available online. Nearly half of the respondents noted that collection materials needed to be viewed onsite at the holding institution per their policy; interestingly, three of those respondents also mentioned that they engage in remote reference services. The discrepancy between these practices may be a definition misunderstanding but highlights that there is more information that can be learned based off this initial study.

This study also found that many of the respondents use redaction as a technique to prepare and/or deliver research materials to students and researchers. Redaction of analog materials is a very manual process which includes photocopying or scanning and then redacting individual words within the document. Digital redaction also must be completed in a way where users cannot break the redaction with their own software. The time-consuming nature of redaction may also put a strain on institutions with small teams who do not have the bandwidth of larger institutions. This is a barrier that may also limit the amount of material that can be accessed by researchers despite having approval to access more.

Further research could identify how common redaction procedures are and in what specific contexts they are employed, as it is hard to reconcile why such a manual process may be so regularly used. Depending on the results of future research, recommendations may be shared that establish standards on the use of redaction and expectations for when it is necessary. If it is possible to reduce instances requiring redaction, it can decrease the burden on staff and increase availability for researchers. In terms of collection materials available digitally, many institutions have some digital material available online. This study specifically asked about online image collections (such as photographs) to capture an aspect of IIHI beyond written documents. Over half of respondents said they do not put images containing patient faces online. While some respondents indicated that they do make images available after the end of HIPAA, this suggests that research materials outside of written documents may be even more restricted due to the inability to completely obscure identities.

The small sample size of this study means that there is limited data to draw from when forming conclusions. Aiming for larger sample sizes in future research would enhance data analysis and offer more opportunities to identify similarities and gaps within access practices. The data collected during this project also cannot be considered representative sample as the number of institutions holding research collections is unknown. Identifying the larger population of research collections is an additional area that can be explored in the future. The questions covered a wide range of topics related to access policies and procedures resulting in a long survey with numerous free-text questions.

Despite efforts to limit the number of free-text questions, the scope of the survey necessitated input directly from respondents to describe complicated access procedures that could not be captured by multiple-choice questions. The length of and time required to complete the survey may have added to the attrition of respondents during the later sets of questions which may have impacted the results discussed in the ‘Other Services’ section. Similarly, using a survey as the single means to gather data and the limited analysis it can yield does not offer the same opportunities as other methods of research. Despite these limitations, this study identified several themes that can inform future research objectives.

### Conclusion and Future Work

This study takes the first step towards gathering information on access practices at various research repositories in the United States. The results offer some preliminary insight into trends and identifies areas of future study. Ideally, future work could eventually lead to guidance on standardizing access policies and creating more uniformity among reference practices for collections that contain historic IIHI. This work could provide evidence for librarians and archivists to advocate for revising access restrictions and reference services at their institutions which would reduce confusion for students and researchers trying to access collections.

The preliminary data and analysis produced by this study set the groundwork for more in-depth research in the future. A more extensive research approach should include a larger sample size targeting a larger number of eligible repositories and focus on recruiting more diverse participants. This study was predominantly completed by archivists and curators and lacks the perspectives of librarians who may also handle these types of requests or staff a special collections department. A targeted and broadly publicized distribution of future surveys or other research methods, such as focus groups, interviews, and policy reviews, may help recruit these participants. Future surveys can be tailored to either address policies or procedures to capture more nuances than were captured during this study, especially relating to rationale behind restrictions beyond the legal limitations. A diversification of research methods, including discussion groups, presentations, or an environmental scan, would also aid in collecting more data – particularly for those questions that failed to receive a follow up during this study.

Access to research collections that contain historic IIHI can be a long and confusing process that is complicated by inconsistent restrictions beyond legal guidelines. Research institutions have to balance ethical practices and patient privacy with the availability of materials to advance research. Librarians, archivists, and curators who facilitate access also have to balance the needs of their parent institutions with the expectations of the communities they serve. These expectations can be confusing and time consuming and difficult to convey to users. Surveying the access practices at various research institutions offers an opportunity to find consistencies that can be used to advocate for more standardized access policies within the professions.

## Data Availability

Data associated with this article cannot be made available due to IRB restrictions.

## SUPPLEMENTAL FILES

**Appendix A:** Survey Instrument

## References

[1] U.S. Department of Health and Human Services. Health Information Privacy. [Internet]. Washington, D.C. [cited 18 June 2025]. https://www.hhs.gov/hipaa/index.html

[2] Department of Health and Human Services (US), Office for Civil Rights. Summary of the HIPAA Privacy Rule. [Internet][rev May 2003, cited 3 Sept 2025]. https://www.hhs.gov/sites/default/files/privacysummary.pdf

[3] Department of Health and Human Services (US), Office for Civil Rights. Summary of the HIPAA Privacy Rule. [Internet][rev May 2003, cited 3 Sept 2025]. https://www.hhs.gov/sites/default/files/privacysummary.pdf

[4] Lawrence, S. Access Anxiety: HIPAA and Historical Research. Journal of the History of Medicine and Allied Sciences. Oct 2007; 62(4): 422–460. https://www.jstor.org/stable/24632368 17204486 10.1093/jhmas/jrl048

[5] IRB Advisor: Rule Creates Headaches for Historical Medical Archives [Internet]. [rev 1 May 2007, cited 3 Sept 2025]. https://www.clinician.com/articles/103378-rule-creates-headaches-for-historical-medical-archives

[6] Department of Health and Human Services (US). Modifications to the HIPAA Privacy, Security, Enforcement, and Breach Notification Rules Under the Health Information Technology for Economic and Clinical Health Act and the Genetic Information Nondiscrimination Act; Other Modifications to the HIPAA Rules. Final rules. Fed Regist. 25 January 2013; 78(17). Available from: https://www.govinfo.gov/content/pkg/FR-2013-01-25/pdf/2013-01073.pdf 23476971

[7] Evans Letocha, P. Recent Changes to the HIPAA Privacy Rule. The Watermark. 2013 Spring; 36(2): 10–19. http://iis-exhibits.library.ucla.edu/alhhs/Watermark_Vol_36_No_2_Spring_2013.pdf

[8] Novak Gustainis E R, Evans Letocha P. The Practice of Privacy. Council on Library and Information Resources; 2015. 21 p. https://www.clir.org/wp-content/uploads/sites/6/ThePracticeofPrivacy.pdf

[9] Holden, J, Roeschley, A. Privacy and Access in the Massachusetts Society for the Prevention of Cruelty to Children Records. American Archivist. 1 Mar 2020; 83(1): 77–90. 10.17723/0360-9081-83.1.77

[10] Galloway, P. Providing Restricted Access to Mental Health Archives Within Government Archives: The Subject Stakeholder. American Archivist. 24 Jun 2021; 84(1): 165–188. 10.17723/0360-9081-84.1.165

[11] Windon, K. Tang, L. Archival Discretion: A Survey on the Theory and Practice of Archival Restrictions. Journal of Contemporary Archival Studies. 2022; 9(11). https://elischolar.library.yale.edu/jcas/vol9/iss1/11

[12] Hawk, A. Reference Staffing and Scheduling Models in Archives and Special Collections: A Survey Analysis of Prepandemic Practices. American Archivist. 28 Dec 2022; 85(2):480–510. 10.17723/2327-9702-85.2.480

[13] NDSA Storage Survey Working Group. 2023 Storage Infrastructure Survey Report [Internet]. Alexandria, VA: National Digital Stewardship Alliance; 14 Aug 2024. [rev 17 Oct 2024, cited 3 Sept 2025]. https://osf.io/9QP4W

[14] Skinner, M. Ioana H. A*CENSUS II All Archivists Survey Report [Internet]. Ithaka S+R; 22 August 2022 [cited 3 Sept 2025]. 10.18665/sr.317224

[15] Qualtrics. [Internet]. Qualtrics [cited 2025 June 16] Available from: https://www.qualtrics.com/

[16] Groves, R. M., Fowler, F. J. J., Couper, M. P., Lepkowski, J. M., Singer, E., & Tourangeau, R. Survey Methodology. 2^nd^ ed. John Wiley & Sons, Incorporated. 2009. p. 2.

[17] Content Analysis. [Internet]. [New York]. Mailman School of Public Health, Columbia University; [cited 2025 June 19]. Available from: https://www.publichealth.columbia.edu/research/population-health-methods/content-analysis

[18] Groves, R. M., Fowler, F. J. J., Couper, M. P., Lepkowski, J. M., Singer, E., & Tourangeau, R. Survey Methodology. 2^nd^ ed. John Wiley & Sons, Incorporated. 2009. 201-207p.

[19] Novak Gustainis E R, Evans Letocha P. The Practice of Privacy. Council on Library and Information Resources; 2015. 21 p. https://www.clir.org/wp-content/uploads/sites/6/ThePracticeofPrivacy.pdf

[20] Dong L, Ilieva P, Medeiros A. Data dreams: planning for the future of historical medical documents. Journal of the Medical Library Association [Internet]. 4 Oct 2018 [cited 23 June 2025]; 106(4). 10.5195/jmla.2018.444 PMC6148606 30271304

[21] Kloss LL, Brodnik MS, Rinehart-Thompson LA. Access and disclosure of personal health information: a challenging privacy landscape in 2016-2018. Yearb Med Inform. 2018 Aug; 27(1):60–66. doi: 10.1055/s-0038-1667071. 30157506 PMC6115206

[22] Walch, V. Part 3: A*Census: A Closer Look Expanded Version [Internet]. Society of American Archivists 2006. [cited 17 Sept 2025]. https://www2.archivists.org/sites/all/files/ACensus-Part3%20Closer%20Look%20-%20Expanded.pdf; Skinner, M. Hulbert, I. A*Census II: All Archivists Survey Report. Society of American Archivists. 21 Aug 2022. [cited 17 Sept 2025]. https://sr.ithaka.org/wp-content/uploads/2022/08/SR-Report-ACENSUS-II-All-Archivists-Survey-08222022.pdf

[23] Some examples include Windon, K. Tang, L. Archival Discretion: A Survey on the Theory and Practice of Archival Restrictions. Journal of Contemporary Archival Studies. 2022; 9(11). https://elischolar.library.yale.edu/jcas/vol9/iss1/11

