# Supplementary material for: Access to historic individually identifiable health information: a multi-institutional survey of research institutions
Source: J Med Libr Assoc. 2026 Jul 14;114(3):238–45. doi: 10.5195/jmla.2026.2259 (PMC13367304; doi:10.5195/jmla.2026.2259)
Supplement: Supplementary file 1 — Appendix A: Survey Instrument [file jmla-114-3-238-s01.pdf]

Survey Instrument

Consent Page

You are invited to participate in an online survey titled "Access to Protected Health Information (PHI)." The purpose of this study is to gather data from medical libraries and archives about their access policies in order to gain a more holistic view across the profession. For the purposes of this survey, PHI is defined as any record that contains health information of an individual. The health information can be in a record of any age, in a HIPAA covered institution or not, and in any format (i.e. documents, photographs, audio, etc).

This online survey should take about 20 to 30 minutes to complete. Participation is voluntary and responses will be kept confidential. Submission of the survey will be interpreted as your informed consent to participate. You have the option to not respond to questions or stop the survey any time you choose. If you have any questions about the research, please contact the Principal Investigator, Amanda Garfunkel, via email at amg4018@med.cornell.edu.

Demographic/General Questions

1. Which category most accurately describes the repository where you work?
(Note: If your repository is a combination of the categories below, please choose the option that most accurately describes the mission of the organization or is most relevant to the unit you work in.)

    Museum
    Corporate/for-profit
    Public/Governmental
    Academic
    Religious
    Medical
    Other:

2. What is the size of your INSTITUTION by employees?

    0-100 people
    100-500 people
    500-1000
    1000-2000
    2000+

3. What is the size of your REPOSITORY by number of staff?
(Note: Please include both full and part-time employees, but do not include interns or volunteers unless they are regularly responsible for reference services)

    1-3
    4-10
    11-20
    21-50
    50+

4. What title most accurately represents your role?

    Librarian

Archivist
Historian
Curator
Records Manager
Other:

5. What is the name of your repository and institution? (Optional):
Free text

6. Are your collections available to the public?
Yes/No

Access to Protected Health Information (PHI) Questions

7. Is your institution a HIPAA covered entity?
Yes/No/Not sure
[If yes, respondents will see Q9-12, if no or not sure respondents will see Q13-16]

8. Are there additional state laws regarding the protection of PHI that you must adhere to?
Yes/No/Not sure
If yes, what state:

9. What are the requirements or procedures for researchers to ACCESS records still covered by HIPAA at your institution?
Free text

10. Is there a point in time when records containing PHI at your institution are freely open for research without prior approvals or other interventions?
Yes, at the end of HIPAA
Yes, a certain number of years after the end of HIPAA
Yes, according to institutional policy beyond HIPAA
No, records always require approval prior to access

11. What is the policy:
Free text

12. If your institution has policies which restrict access to records containing PHI after the end of HIPAA, when are access exemptions made? For example, requests from descendants, PHI will be redacted by the researcher, etc.
Free text

13. Do you allow access to records containing PHI if the individual is most likely still living?
Yes/No/Other

14. Is there a point in time when records containing PHI at your institution are freely open for research without prior approvals or other interventions?
Yes, we have a set institutional policy
Yes, it’s under the discretion of the department head

No, records always require approval prior to access

15. If it’s an institutional policy, please explain:
Free text

16. If your institution has policies which restrict access to records containing PHI when are access exemptions made? For example, requests from descendants, PHI will be redacted by the researcher, etc.
Free text

17. Do you ever use redaction in order to provide access to records containing PHI?
Yes/No

18. If yes, please explain how:
Free text

Use of PHI Records
(Note: for this section the term ‘after HIPAA’ can also refer to after your institution’s restriction period.)

19. If access is granted to records containing PHI, are they only available to be viewed onsite?
Yes/No/Yes, only after HIPAA/Other

20. What uses of historic PHI records (after HIPAA or individuals are believed to be deceased) require prior approval or other interventions, if any?
Identified data
Publication
Photocopies/Photographs
No prior approval or interventions needed
Other:

21. Do you ever provide remote reference services for records containing PHI?
(Note: Remote reference services may include sending reproductions to researchers, use of a virtual terminal, or orally conveying information, etc.)
Yes/No/Yes, only after HIPAA/Other

If yes, please explain:
Free text

23. Does your repository put digitized records containing PHI or photographs with patient faces online?
Yes/No/Yes, only after HIPAA/Other

Documentation

24. Can you briefly summarize your access policy to records containing PHI or provide a link if it’s publicly available?
Free text

25. When was the last time you updated your access policy (if applicable)?
1-2 years ago
2-5 years ago
5-7 years ago
7+ years ago
We're in the process of updating it now
Not applicable

26. How are access policies for records containing PHI communicated to researchers?
Access policy and potential restrictions are available online
Access process is communicated directly to the researcher (phone, email, etc.)
Restrictions are listed in guides/finding aids for individual collections
Other:

Conclusion

27. Is there anything else you'd like to share about your institution's access policies for collections with PHI?
Free text

28. Would you be willing to be contacted in the future for any follow-up questions?
Yes/No

29. If yes, what is the best way to contact you?
Free text
